# Blood pressure management in patients receiving rescue stenting after failed endovascular treatment in large vessel occlusion acute ischaemic stroke: a multicentre registry

**DOI:** 10.1093/esj/aakag035

**Published:** 2026-05-11

**Authors:** Aikaterini Anastasiou, Alex Brehm, Johannes Kaesmacher, Adnan Mujanovic, Marta de Dios Lascuevas, Tomas Carmona, Alfonso López-Frías, Blanca Hidalgo Valverde, Thanh N Nguyen, Mohamad Abdalkader, Piers Klein, Guillaume Thevoz, Patrik Michel, Bruno Bartolini, Marius Kaschner, Daniel Weiss, Andrea M Alexandre, Alessandro Pedicelli, Paolo Machi, Gianmarco Bernava, Shuntaro Kuwahara, Kazutaka Uchida, Jason Wenderoth, Anirudh Joshi, Grzegorz Karwacki, Manuel Bolognese, Agostino Tessitore, Sergio Lucio Vinci, Amedeo Cervo, Claudia Rollo, Ferdinand Hui, Aaisha Siddiqua Mozumder, Daniele Giuseppe Romano, Giulia Frauenfelder, Nitin Goyal, Vivek Batra, Violiza Inoa-Acosta, Christophe Cognard, Matúš Hoferica, Riitta Rautio, Daniel P O Kaiser, Johannes C Gerber, Julian Clarke, Michael R Levitt, Marcel N Wolf, Ahmed E Othman, Timo Uphaus, Luca Scarcia, Erwah Kalsoum, Diana Melancia, Diana Aguiar de Sousa, Maria Porzia Ganimede, Vittorio Semeraro, Flavio Giordano, Massimo Muto, Umesh Bonala, Anil M Tuladhar, Sjoerd F M Jenniskens, Victoria Hellstern, Ilka Kleffner, Paolo Remida, Susanna Diamanti, Leonardo Renieri, Elena Ballabio, Luca Valvassori, Wiesmann Martin, Frederic De Beukelaer, Vera Aebischer, Dang Hoang-Tho, Nikki Rommers, Mira Katan, Aristeidis H Katsanos, Marios-Nikos Psychogios

**Affiliations:** Department of Neuroradiology, University Hospital Basel, Basel, Switzerland; Department of Neuroradiology, University Hospital Basel, Basel, Switzerland; Department of Diagnostic and Interventional Neuroradiology, Inselspital University Hospital Bern, University of Bern, Bern, Switzerland; Diagnostic and Interventional Neuroradiology, CIC-IT 1415, CHRU de Tours, Tours, France; Department of Diagnostic and Interventional Neuroradiology, Inselspital University Hospital Bern, University of Bern, Bern, Switzerland; Interventional Neuroradiology, Vall d'Hebron University Hospital, Barcelona, Spain; Interventional Neuroradiology, Vall d'Hebron University Hospital, Barcelona, Spain; Interventional Neuroradiology, Hospital Clínico San Carlos, Madrid, Spain; Stroke Unit, Department of Neurology, Hospital Clínico San Carlos, Madrid, Spain; Department of Radiology (M.A., T.N.N., P.K.), Boston Medical Center, Boston University Chobanian & Avedisian School of Medicine, Boston, MA, United States; Department of Radiology (M.A., T.N.N., P.K.), Boston Medical Center, Boston University Chobanian & Avedisian School of Medicine, Boston, MA, United States; Department of Radiology (M.A., T.N.N., P.K.), Boston Medical Center, Boston University Chobanian & Avedisian School of Medicine, Boston, MA, United States; Stroke Center, Neurology Service, Department of Clinical Neurosciences, Lausanne University Hospital, Lausanne, Switzerland; Stroke Center, Neurology Service, Department of Clinical Neurosciences, Lausanne University Hospital, Lausanne, Switzerland; Stroke Center, Neurology Service, Department of Clinical Neurosciences, Lausanne University Hospital, Lausanne, Switzerland; Department of Diagnostic and Interventional Radiology, Medical Faculty and University Hospital Düsseldorf, Heinrich-Heine-University Düsseldorf, Düsseldorf, Germany; Department of Diagnostic and Interventional Radiology, Medical Faculty and University Hospital Düsseldorf, Heinrich-Heine-University Düsseldorf, Düsseldorf, Germany; UOSA Neuroradiologia Interventistica, Fondazione Policlinico Universitario A.Gemelli IRCCS, Roma, Italy; UOSA Neuroradiologia Interventistica, Fondazione Policlinico Universitario A.Gemelli IRCCS, Roma, Italy; Università Cattolica del Sacro Cuore, UOSA Neuroradiologia Interventistica, Roma, Italy; Division of Neuroradiology, Geneva University Hospitals, Geneva, Switzerland; Division of Neuroradiology, Geneva University Hospitals, Geneva, Switzerland; Department of Neurosurgery, Hyogo Medical University, Nishinomiya, Japan; Department of Neurosurgery, Hyogo Medical University, Nishinomiya, Japan; Institute of Neurological Sciences, Prince of Wales Hospital, Sydney, NSW, Australia; Prince of Wales Clinical School, University of New South Wales, Sydney, Australia; Institute of Neurological Sciences, Prince of Wales Hospital, Sydney, NSW, Australia; Prince of Wales Clinical School, University of New South Wales, Sydney, Australia; Department of Radiology and Nuclear Medicine, Cantonal Hospital Lucerne, Lucerne, Switzerland; Neurocenter, Cantonal Hospital of Lucerne, Lucerne, Switzerland; Neuroradiology Unit, University Hospital A.O.U. "G. Martino" - Messina, Messina, Italy; Department of Biomedical, Dental and Morphological and Functional Imaging (BIOMORF), University of Messina, Messina, Italy; Department of Neuroradiology, Grande Ospedale Metropolitano Niguarda, Milan, Italy; Department of Neuroradiology, Grande Ospedale Metropolitano Niguarda, Milan, Italy; Department of Neuroradiology, Grande Ospedale Metropolitano Niguarda, Milan, Italy; Neuroscience Institute, The Queen’s Medical Center, Honolulu, HI, USA; University of Hawaii, Neuroscience Institute, Honolulu, HI, United States; Neuroscience Institute, The Queen’s Medical Center, Honolulu, HI, USA; University of Hawaii, Neuroscience Institute, Honolulu, HI, United States; Unit of Interventional Neuroradiology, University Hospital AOU Salerno, Italy; Unit of Interventional Neuroradiology, University Hospital AOU Salerno, Italy; Department of Neurology, University of Tennessee Health Science Center, Memphis, TN, United States; Department of Neurological Surgery, Semmes-Murphey Clinic, Memphis, TN, United States; Department of Neurology, University of Tennessee Health Science Center, Memphis, TN, United States; Department of Neurological Surgery, Semmes-Murphey Clinic, Memphis, TN, United States; Department of Neurology, University of Tennessee Health Science Center, Memphis, TN, United States; Department of Neurological Surgery, Semmes-Murphey Clinic, Memphis, TN, United States; Neuroradiology Department, Toulouse University Hospital, INSERM, U1048 and Université Toulouse 3, I2MC,Toulouse, France; Neuroradiology Department, Toulouse University Hospital, INSERM, U1048 and Université Toulouse 3, I2MC,Toulouse, France; Turku University Hospital and University of Turku, Turku, Finland; Institute of Neuroradiology, University Hospital Carl Gustav Carus, Technische Universität Dresden, Dresden, Germany; Dresden Neurovascular Center, Faculty of Medicine and University Hospital Carl Gustav Carus, Technische Universität Dresden, Dresden, Germany; Institute of Neuroradiology, University Hospital Carl Gustav Carus, Technische Universität Dresden, Dresden, Germany; Dresden Neurovascular Center, Faculty of Medicine and University Hospital Carl Gustav Carus, Technische Universität Dresden, Dresden, Germany; Department of Neurological Surgery, University of Washington, Seattle, WA, United States; Department of Neurological Surgery, University of Washington, Seattle, WA, United States; Department of Neuroradiology, University Medical Center Mainz, Johannes Gutenberg University, Mainz, Germany; Department of Neuroradiology, University Medical Center Mainz, Johannes Gutenberg University, Mainz, Germany; Department of Neurology, University Medical Center Mainz, Mainz, Germany; Department of Neuroradiology, Henri Mondor Hospital, Créteil, France; Department of Neuroradiology, Henri Mondor Hospital, Créteil, France; Stroke Center, Department of Neurosciences, Lisbon Central University Hospital - ULS São José, Portugal; Institute of Pharmacology and Neurosciences, Faculty of Medicine, University of Lisbon, Lisbon, Portugal; Faculdade de Medicina, Universidade de Lisboa and Gulbenkian Institute for Molecular Medicine, Lisbon, Portugal; Stroke Center, Department of Neurosciences, Lisbon Central University Hospital - ULS São José, Portugal; Institute of Pharmacology and Neurosciences, Faculty of Medicine, University of Lisbon, Lisbon, Portugal; AORN CARDARELLI, Naples, Italy; Diagnostic and Interventional Neuroradiology, SS. Annunziata Hospital, Taranto, Italy; AORN CARDARELLI, Naples, Italy; AORN CARDARELLI, Naples, Italy; Division of Neurology, McMaster University and Population Health Research Institute, Hamilton, ON, Canada; Department of Neurology, Radboud University Medical Center, Nijmegen, The Netherlands; Donders Institute for Brain, Cognition and Behaviour, Radboud University, Nijmegen, The Netherlands; Department of Radiology & Nuclear Medicine, Radboud University Medical Center, Nijmegen, The Netherlands; Klinikum Stuttgart, Stuttgart, Germany; Stroke Medicine and Neurocritical Care Palliative Care, Neurovascular Ultrasound, Knappschaft Kliniken, University Hospital Bochum, Bochum, Germany; Neuroradiology - Fondazione IRCCS San Gerardo dei Tintori, Monza, Italy; Neurology - Fondazione IRCCS San Gerardo dei Tintori, Monza, Italy; Neurovascular Interventional Unit, Careggi University Hospital, Florence, Italy; Department of Neurology, ASST Santi Paolo e Carlo, Milan, Italy; Department of Neuroradiology, ASST Santi Paolo e Carlo, Milan, Italy; Department of Diagnostic and Interventional Neuroradiology, University Hospital RWTH Aachen, Aachen, Germany; Department of Diagnostic and Interventional Neuroradiology, University Hospital RWTH Aachen, Aachen, Germany; Department of Neuroradiology, University Hospital Basel, Basel, Switzerland; Department of Neuroradiology, University Hospital Basel, Basel, Switzerland; Department of Clinical Research, University of Basel, University Hospital Basel, Basel, Switzerland; Department of Neurology, Stroke Center, University and University Hospital of Basel, Basel, Switzerland; Division of Neurology, McMaster University and Population Health Research Institute, Hamilton, ON, Canada; Department of Neuroradiology, University Hospital Basel, Basel, Switzerland

**Keywords:** ischaemic stroke, thrombectomy, blood pressure management, rescue stenting, intracranial atherosclerotic disease

## Abstract

**Objectives:**

Rescue stenting (RS) has emerged as a bailout strategy after failed reperfusion during endovascular treatment (EVT). Optimal blood pressure (BP) management after RS remains unclear. Our aim is to evaluate the association of BP levels and blood pressure variability (BPV) during the first 24 h after RS with short-term and long-term patient outcomes.

**Methods:**

We performed a retrospective analysis of an international registry where data from adult patients who underwent either RS or rescue angioplasty after failed EVT were collected. Patients who received RS with large vessel occlusion and at least 4 BP measurements in the first 24 h were included.

**Results:**

RS was performed in 437 patients (40.5% female, mean age 67.1 ± 13 years). Admission median National Institutes of Health Stroke Scale score was 12 (IQR 7–18) and history of hypertension was present in 74.2% of patients. Μean Systolic BP (SBP) in the first 24 h was 137.4 ± 14.6 mmHg. Higher values of BPV (coefficient of variation, standard deviation, average real variability and successive variation) were associated with lower odds for Modified Rankin Scale score 0–2 at 90 days (adjusted odds ratio ranging 0.55 [0.38, 0.79] to 0.99 [0.98, 0.99] per 10 units increase). No associations were found between any SBP measure and death, sICH as well as neurological deterioration at 24 h.

**Conclusion:**

In our study, higher BPV was associated with worse clinical outcomes in stroke patients treated with RS as bailout therapy after failed reperfusion. No association was shown between mean, maximum, minimum and delta SBP and clinical outcomes.

## Graphical Abstract

**Graphical Abstract:**
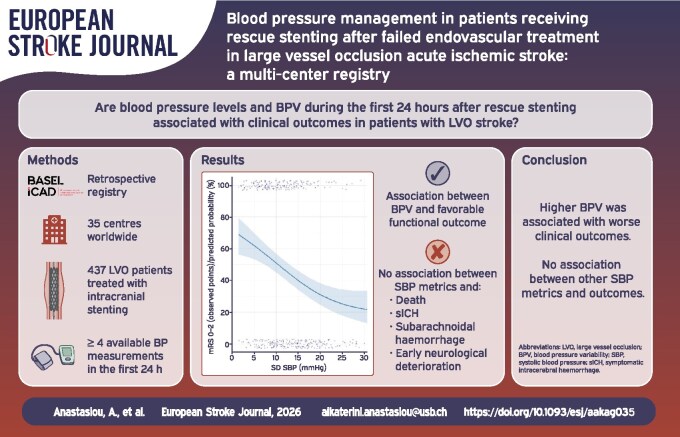

## Introduction

In 10%–20% of acute ischaemic stroke patients, endovascular treatment (EVT) fails to achieve successful reperfusion with most cases attributed to intracranial atherosclerotic disease (ICAD).^1–4^ In those cases, during EVT often even after initial successful reperfusion, the vessel reoccludes within minutes. It is suspected that re-thrombosis occurs from an activated atherosclerotic plaque. As failed reperfusion has been associated with an increased risk of early neurologic deterioration and worse outcomes,^5,6^ rescue stenting (RS) has emerged as a bailout strategy in failed EVT cases.^7–12^

The optimal BP target in the first 24 h after EVT remains uncertain. High BP is common post-EVT and has been associated to greater risks of haemorrhagic transformation, cerebral oedema and poor outcomes.^13,14^ However, excessive BP lowering may reduce collateral perfusion.^15^ These issues may be more relevant in patients requiring RS. In this group, clinicians must balance the need for more permissive BP to avoid hypoperfusion against the increased risk of haemorrhage due to antiplatelet therapy. Current guidelines recommend maintaining systolic BP (SBP) < 180 mmHg, however whether different targets are needed after RS is unknown.^16^ Beyond absolute BP values, growing attention has been directed towards blood pressure variability (BPV), the degree of fluctuation in BP over time, which appears to be independently associated with poor clinical outcomes.^17^

This study aims to investigate possible associations between BP levels during the first 24 h after RS in large vessel occlusion (LVO) cases and the clinical outcomes of patients.

## Methods

In this retrospective cohort study, we performed a post-hoc analysis of the blood pressure (BP) and Antiplatelet medication management after reScue angioplasty after failed EVT in large and distal vessel occlusions with probable IntraCranial Atherosclerotic Disease (BASEL ICAD) registry.^18,19^ In this international registry, 35 centres collected data of adult patients who underwent either RS or rescue angioplasty after failed EVT between 1 January 2019 and 31 December 2023. Data from participating centres were curated by reviewing patient charts and procedure notes. The registry was approved by the applicable ethics committee (BASEC ID 2024-00904).

For this sub-analysis of the BASEL ICAD registry, only patients with LVO who received RS were included (*n* = 608). Possible underlying ICAD was deemed by the treating physician. In 8 centres, BP was measured using a central arterial line and with a conventional non-invasive BP cuff in 12 centres. We excluded all patients with less than 4 BP measurements over the first 24 h (*n* = 171). The analysis set used in the present work consists of 437 patients. (Figure S1). The number of BP measurements per patient is displayed in Table S1. Primary outcome of interest was functional independence, defined as a modified Rankin Scale (mRS) Score 0–2 at 90 days. Primary safety outcome of interest was the all-cause mortality within 90 days. Secondary outcomes of interest included early neurological deterioration, defined by increase in the National Institute of Health Stroke Scale (NIHSS) score >2 at 24 h, symptomatic intracranial haemorrhage (sICH) and any subarachnoid haemorrhage (SAH).

### Blood pressure variables

We considered different variables related to the central point or variance of SBP in the first 24 h after RS. All respective variables were calculated from the available number of SBP measures per patient (ie, at least four measurements). To indicate the central value, we considered the mean SBP, maximum SBP, minimum SBP, delta SBP (ie, the difference between the minimum and maximum SBP). As measures of variation, we considered the standard deviation (SD) of all SBP measures, the coefficient of variation (CV), successive variation (SV) (square root of the mean squared successive difference) and the average real variability (ARV) (mean of successive differences) of SBP measures.

### Statistical analysis

Baseline and stroke characteristics are summarised as mean (SD) or median (interquartile range [IQR]) for continuous variables and frequency (percentage) for categorical variables. For the SBP variables, we additionally provide the range.

Associations between SBP variables and outcomes were estimated using logistic regression models, adjusted for a set of pre-defined confounders. Pre-defined confounders were age, sex, pre-stroke mRS (0–2 vs 3–5), admission NIHSS, intravenous thrombolysis (IVT) (yes/no) and occlusion territory (anterior vs posterior circulation). To account for clustering by centre, all models included centre as a random intercept.

For each SBP variable-outcome pair, we first examined whether the relationship between the SBP variable and the log odds of the outcome was linear or non-linear. This was assessed visually by fitting a locally estimated scatterplot smoothing (LOESS) curve to the partial residuals of the regression model. We then compared model fit between a standard linear specification and restricted cubic splines with two and three degrees of freedom using the Akaike Information Criterion (AIC). When splines improved fit, we also evaluated quadratic polynomial. If the quadratic model fit was comparable to the best-fitting spline according to AIC, we preferred the quadratic model for interpretability; otherwise, the spline specification was retained.

Results are reported as adjusted odds ratios (aORs) with 95% profile-likelihood confidence intervals and *P*-values. Given the retrospective design of this study, *P*-values were not adjusted for multiple comparisons. All analyses used available cases, and standard model diagnostics (influence, leverage, multicollinearity) were examined and deemed acceptable.

To assess potential bias due to hourly BP registration being available for some patients but not others, we compared baseline characteristics of patients included in this analysis (≥four hourly measurements) with those with fewer measurements. As a second sensitivity analysis, we also evaluated the association between the total number of SBP measurements over 24 h and all outcomes to explore possible bias towards more intensive monitoring in higher-risk patients. To address potential selection bias due to incomplete hourly BP registration, we performed 2 sensitivity analyses: repeating the analysis in patients with at least 2 rather than 4 BP measurements and applying inverse probability of inclusion weighting based on baseline and stroke-related characteristics. Details of the sensitivity analyses are provided in the Supplementary material.

Statistical analyses were performed in R v4.4.1 (https://www.r-project.org/). Reporting follows the STROBE guidelines (http://www.strobestatement.org).

## Results

Overall, 437 patients met the inclusion criteria (40.5% female, mean age 67.1 ± 13 years). History of hypertension was reported in 74.2% of the cases. The median baseline NIHSS was 12 (IQR 7–18). IVT was administered prior to EVT in 24.1% of patients. The median time from symptom onset to groin puncture was 339 (IQR 203.5–620.5) minutes. After a median of two EVT passes (IQR 1–3), RS was performed. Median time from onset to reperfusion was 436 min (IQR 291.8–719.5). A complete overview of the baseline characteristics is displayed in Table 1. Overall, 44.8% of patients reached functional independence at 90 days (mRS 0–2). Mortality at 90 days was 25.4%. Postprocedural stent occlusion occurred in 51 patients (12.7%), with 62.2% of these occlusions within 24 h. In 8.1% of patients (*n* = 34), sICH was reported and 80 (19.9%) experienced any SAH. The median NIHSS at 24 h was 8 (IQR 3–17). Table S2 summarises the outcomes.

**Table 1:** Patient characteristics.

Baseline characteristics	**All patients (*n* = 437)** ^**a**^	mRS 0–2 (*n* = 182)	mRS 3–6 (*n* = 224)	*P*-value
**Age**	67.1 (13.0)	64.4 (13.8)	69.2 (12.0)	<.001
**Sex = Male**	260 (59.5)	118 (64.8)	124 (55.4)	.067
**Hypertension**	313 (74.2)	127 (72.2)	164 (75.9)	.464
**Dyslipidemia**	142 (40.0)	69 (43.9)	66 (37.5)	.278
**Diabetes mellitus**	140 (33.5)	60 (34.1)	71 (33.2)	.934
**Coronary artery occlusive disease**	65 (16.5)	29 (17.4)	35 (17.2)	1.000
**Current or past smoking**	145 (37.0)	67 (40.6)	68 (34.0)	.233
**Atrial fibrillation**	61 (14.7)	23 (13.1)	35 (16.4)	.433
**History of stroke or TIA**	103 (25.7)	45 (27.3)	50 (24.3)	.590
**Pre-stroke mRS**				
** 0**	312 (75.7)	151 (84.8)	145 (69.4)	
** 1**	55 (13.3)	19 (10.7)	32 (15.3)	
** 2**	24 (5.8)	6 (3.4)	14 (6.7)	
** 3**	17 (4.1)	2 (1.1)	14 (6.7)	
** 4**	4 (1.0)	0 (0.0)	4 (1.9)	
** 5**	0 (0.0)	0 (0.0)	0 (0.0)	
**Race**				.256
** White**	308 (76.8)	135 (77.6)	156 (77.6)	
** Asian**	27 (6.7)	15 (8.6)	9 (4.5)	
** African American**	57 (14.2)	21 (12.1)	31 (15.4)	
** Native Hawaian or pacific**	8 (2.0)	2 (1.1)	5 (2.5)	
** American Indian**	1 (0.2)	1 (0.6)	0 (0.0)	
**Stroke characteristics**				
**NIHSS admission**	12.0 [6.8, 18.0]	14.5 [8.0, 20.0]	9.0 [5.0, 14.0]	<.001
**IVT**	105 (24.1)	47 (21.1)	49 (27.1)	.197
**Time onset to groin puncture (min)**	339.0 [203.5, 620.5]	365.0 [208.5, 674.5]	300.0 [197.0, 582.0]	.145
**Occluded vessel**				
** BA**	93 (21.4)	60 (26.8)	31 (17.0)	
** ICA-T**	64 (14.7)	29 (12.9)	32 (17.6)	
** M1**	243 (56.0)	116 (51.8)	108 (59.3)	
**Intervention characteristics**				
**Anaesthesia = Sedation**	136 (36.6)	66 (34.6)	60 (39.0)	.464
**Highest mTICI achieved before stenting**				.133
** 0**	87 (21.9)	51 (24.9)	28 (16.9)	
** 1**	69 (17.3)	30 (14.6)	35 (21.1)	
** 2a**	62 (15.6)	35 (17.1)	22 (13.3)	
** 2b**	105 (26.4)	56 (27.3)	45 (27.1)	
** 2c**	29 (7.3)	16 (7.8)	13 (7.8)	
** 3**	46 (11.6)	17 (8.3)	23 (13.9)	
**mTICI final pass**				.025
** 0**	12 (2.8)	10 (4.5)	2 (1.1)	
** 1**	8 (1.9)	5 (2.3)	1 (0.6)	
** 2a**	18 (4.2)	11 (5.0)	6 (3.3)	
** 2b**	128 (29.7)	68 (30.9)	53 (29.3)	
** 2c**	68 (15.8)	40 (18.2)	23 (12.7)	
** 3**	197 (45.7)	86 (39.1)	96 (53.0)	
**Number of passes**	2.0 [1.0, 3.0]	3.0 [2.0, 4.0]	2.0 [1.0, 3.0]	.004
**Time onset to reperfusion (min)**	436.0 [291.8, 719.5]	489.5 [319.0, 762.8]	382.0 [282.0, 678.0]	.117
**Aetiology rescue stenting**				.768
** ICAD**	388 (91.1)	199 (92.1)	164 (90.6)	
** Dissection**	33 (7.7)	15 (6.9)	14 (7.7)	
** Hard thrombus**	5 (1.2)	2 (0.9)	3 (1.7)	
**Complications**				.208
** No**	329 (81.8)	165 (80.5)	148 (86.0)	
** SAH/vessel perforation**	19 (4.7)	11 (5.4)	6 (3.5)	
** Dissection**	10 (2.5)	5 (2.4)	5 (2.9)	
** Femoral/retroperitoneal hematoma**	9 (2.2)	3 (1.5)	5 (2.9)	
** Other**	35 (8.7)	21 (10.2)	8 (4.7)	

Abbreviations: BA = basilar artery; ICAD = intracranial atherosclerotic disease; ICT-T = internal carotis terminal; IVT = intravenous thrombolysis; mRS = Modified Rankin Scale; mTICI = modified thrombolysis in cerebral infarction; M1 = M1 segment of the middle cerebral artery; NIHSS = National Institutes of Health Stroke Scale; SAH = subarachnoid haemorrhage; TIA = transient ischaemic attack.

^a^Thirty patients without documented mRS at 90 days.

### BP measurements and associations

**Figure 1:**
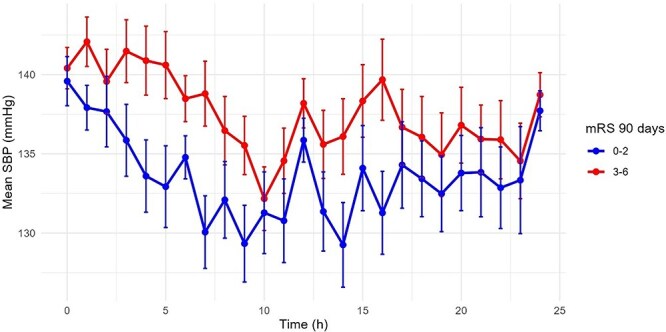
Mean systolic blood pressure (SBP) in mmHg with standard error (SE) (error bars) over the first 24 h by outcome group (mRS 0-2 and 3-6 at 90 days).

The course of SBP over 24 h for all patients is displayed in Figure 1, stratified by functional independence at 90 days (yes/no). Mean SBP within the first 24 h post-EVT was 137.4 (SD 14.6) mmHg. SBP SD was 13.8 (7.4) mmHg, SBP CV was 10.2 (5.7), ARV in SBP was 13.1 (8.3) and SV SBP was 16.1 (9.6). The median, quartiles, minimum and maximum values for each of the variables are presented in Table S3.

**Figure 2:**
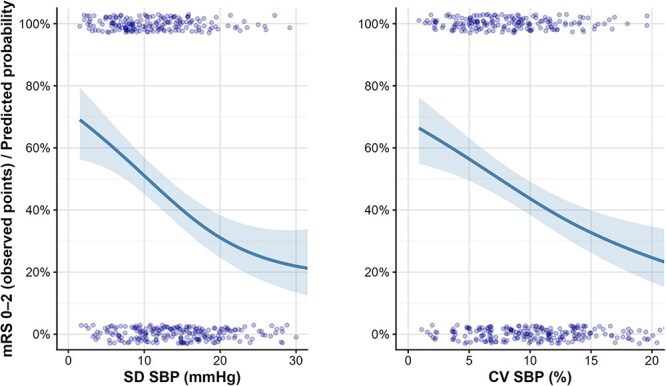
Association between systolic blood pressure (SBP) variability and favourable functional outcome. Scatter points show individual patients with favourable outcome (mRS 0–2; coded 1) or unfavourable outcome (mRS 3–6; coded 0) with slight vertical jitter for visibility. The solid line depicts the estimated probability of mRS 0–2 at 90 days across the range of SBP variability measures. Left panel: SBP standard deviation (SD, mmHg). Right panel: SBP coefficient of variation (CV, %). Higher SBP variability is associated with a lower estimated probability of mRS 0–2.

Figure 2 shows probability of functional independence at 90 days across the entire range of SBP measures. After model evaluations, linear models were fit for all pairs of SBP variable and outcome, except maximum SBP with outcome mRS 0–2 and sICH. Here a quadratic polynomial model resulted in the best fit. After adjustment for all the predefined confounders, higher values of variation in SBP (CV, SD, ARV and SV) were associated with lower odds for mRS 0–2 at 90 days (Table 2). We found no evidence of an association between any SBP measure and death, symptomatic intracerebral haemorrhage (sICH), subarachnoid haemorrhage (SAH) or early neurological deterioration defined as an increase in NIHSS >2 at 24 h. In other words, after adjustment for relevant clinical factors, none of the SBP summary or variability metrics appeared to be associated with these early safety or clinical worsening endpoints. Adjusted estimates for these secondary outcomes are provided in Table S3. Details about both sensitivity analyses are included in the Supplementary material.

**Table 2:** Adjusted BP association with functional independence at 90 days (mRS 0-2).

	aOR	95% CI	*P*-value
**mRS 0-2 at 90 days**			
**Mean SBP (per 10 mmHg)**	0.84	0.71–0.99	.046
**Median SBP (per 10 mmHg)**	0.84	0.71–0.99	.046
**Maximum SBP (per 10 mmHg)**	0.86	0.73–1.00	.063
**(Maximum SBP (per 10 mmHg), quadratic term)**	0.91	0.80–1.04	.176
**Minimum SBP (per 10 mmHg)**	1.00	1.00–1.00	.280
**Maximum difference in SBP over 24 h (per 10 mmHg)**	1.02	0.90–1.16	.738
**Standard deviation SBP over 24 h (per 10 mmHg)**	0.87	0.79–0.96	.007
**Coefficient of variation SBP over 24 h (per 0.1)**	0.55	0.38–0.79	.002
**Average real variability SBP (per 10 mmHg)**	0.99	0.98–0.99	.004
**Successive variation SBP (per 10 mmHg)**	0.59	0.41–0.81	.002

Abbreviations: aOR = adjusted odds ratio per one unit increase; CI = confidence interval; mRS = Modified Rankin Scale; SBP = systolic blood pressure.

## Discussion

In this post-hoc analysis of a retrospective multicentre cohort, we analysed 437 patients with LVO who received RS and investigated the association of BP parameters during the first 24 h with functional outcomes. We found that higher BPV was significantly associated with worse clinical outcomes at 90 days. No association was shown between mean, maximum, minimum and delta SBP and clinical outcomes. To the best of our knowledge, this study is the first to report associations of BP parameters with clinical outcomes after RS in LVO cases.

The baseline characteristics and outcomes in our study align with other retrospective studies of RS.^7–12^ Notably, our population had a higher prevalence of hypertension (74% vs. 56%), diabetes (33% vs. 13% and 14.6%) and prior history of stroke (25.7% vs. 12.4%), whereas lower prevalence of atrial fibrillation (14.7% vs. 28.3%), compared to cohorts of unselected acute LVO population undergoing EVT.^1,20^ In our study, 91.1% of the physicians suspected ICAD as the underlying cause of the stenosis. The ICAD population represents a distinct subgroup compared to the average LVO population, as they have more atherosclerotic risk factors. Thus, the higher rates of male sex, hypertension, dyslipidemia, diabetes mellitus and posterior circulation occlusions in our population. Patients undergoing RS are often considered at increased risk of bleeding due to the need for antiplatelet loading followed by continuous antiplatelet maintenance.^20^ Despite this, in our study we report sICH of 9%, similar to previous reports.^7,12,20^

Regarding the mean SBP, a meta-analysis of unselected patients undergoing EVT reported that higher mean SBP levels during the first 24 h after EVT were associated with a lower likelihood of functional improvement.^21^ In our study, we could not show a statistically significant correlation, although we do see a trend towards worse outcomes as the mean SBP level increases (Figure 1).

In our analysis, we were able to show that higher BPV values, including SBP CV, SBP SD, ARV and SV, were significantly associated with worse clinical outcomes at 90 days. Our findings are consistent with a previous meta-analysis of unselected LVO population undergoing EVT.^17^ Furthermore, in our study higher BPV values were not associated with higher risk of sICH, which is also consistent with the current literature.^17^ These findings suggest that BPV also in the RS population may play a significant role in long-term outcomes, underscoring the need for careful BP monitoring and management in stroke patients with RS after failed reperfusion during EVT. Impaired cerebral autoregulation, haemodynamic instability and a potentially increased bleeding risk, although not observed in our dataset, in the setting of RS and concomitant antiplatelet therapy may contribute to the observed association. However, given the retrospective design of our study, these findings should not be interpreted as causal but rather as associative, and BPV may represent either a contributing factor or an epiphenomenon reflecting overall clinical instability. Further investigation is needed to explore the underlying mechanisms and potential therapeutic strategies to mitigate BPV in this population.

This study has limitations, including its non-randomised retrospective design. The cohort may be underpowered to detect existing associations, for example, between mean SBP and clinical outcomes. As we excluded all patients with less than four SBP measurements, there might be selection bias, because of the possibility that patients with more comorbidities or complications are closely monitored and have more SBP measurements. Four measurements were used as a pragmatic minimum to allow estimation of sequential variability indices, but this is lower than would be ideal for a stable assessment of 24-h BP variability. Therefore, the BP findings should be interpreted as exploratory and hypothesis-generating. Furthermore, the combination of various peri- and postinterventional antihypertensive medications and BP management protocols leads to heterogeneity in the study population. Finally, the lack of certain follow-up imaging information, particularly infarct volume assessment, as well as the limited number of available BP measurements (in some cases only four), represent additional limitations of this study.

## Conclusion

Higher BPV was associated with worse clinical outcomes in acute ischaemic stroke patients treated with RS as bailout therapy after failed reperfusion during EVT. No association was shown between mean, maximum, minimum and delta SBP and clinical outcomes.

## Supplementary Material

aakag035_Supplementary_material
