# Supplementary material for: Blood pressure management in patients receiving rescue stenting after failed endovascular treatment in large vessel occlusion acute ischaemic stroke: a multicentre registry
Source: Eur Stroke J. 2026 May 11;11(5):aakag035. doi: 10.1093/esj/aakag035 (PMC13160419; doi:10.1093/esj/aakag035)
Supplement: aakag035_Supplementary_material [file aakag035_supplementary_material.zip › BASEL_ICAD_BP_Supplement.docx]

**Blood pressure management by rescue stenting after failed endovascular treatment in large vessel occlusion acute ischemic stroke: A large retrospective multi-center registry**

**Supplemental material.**

1. Sensitivity analysis

To assess potential bias due to hourly BP registration being available for some patients but not others, we compared baseline characteristics of patients included in this analysis (≥four hourly measurements) with those with fewer measurements. This first sensitivity analysis showed a higher proportion of patients with dyslipidemia in the patients with four or more SBP measurements, compared to patients with less than four SBP measurements. The distribution of the pre-stroke mRS is different, but a comparable proportion of patients had a pre-stroke mRS of 0–1 (Supplemental table 4).

As a second sensitivity analysis, we also evaluated the association between the total number of SBP measurements over 24 hours and all outcomes to explore possible bias toward more intensive monitoring in higher-risk patients. No association was found between the number of available SBP measurements and any of the outcomes.

**Supplement figure 1:** Flow chart


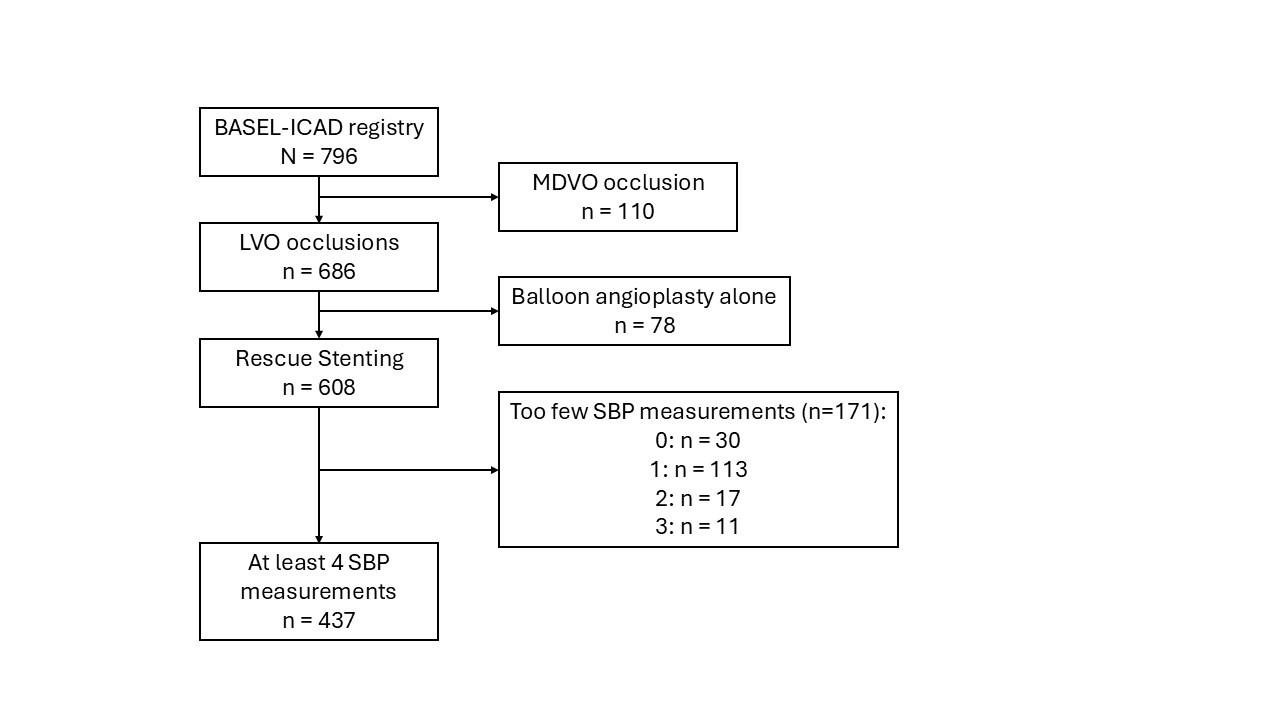


| **Number of measurements** | **Patients (n (%))** |
| --- | --- |
| 0 | 30 (4.9) |
| 1 | 113 (18.6) |
| 2 | 17 (2.8) |
| 3 | 11 (1.8) |
| 4 | 37 (6.1) |
| 5 | 144 (23.7) |
| 6 | 10 (1.6) |
| 7 | 37 (6.1) |
| 8 | 4 (0.7) |
| 9 | 4 (0.7) |
| 10 | 2 (0.3) |
| 11 | 5 (0.8) |
| 13 | 9 (1.5) |
| 14 | 9 (1.5) |
| 15 | 12 (2) |
| 16 | 10 (1.6) |
| 17 | 3 (0.5) |
| 18 | 2 (0.3) |
| 19 | 8 (1.3) |
| 20 | 2 (0.3) |
| 21 | 5 (0.8) |
| 22 | 5 (0.8) |
| 23 | 9 (1.5) |
| 24 | 24 (3.9) |
| 25 | 96 (15.8) |

**Supplement table 1:** Measurements from hour 0 to hour 24. The table displays a summary of the total number of hourly measurements per patient with percentage.

| **Outcomes** | **All patients (n=437)** |
| --- | --- |
| mRS 90 days |  |
| 0 | 61 (15.0) |
| 1 | 70 (17.2) |
| 2 | 51 (12.6) |
| 3 | 43 (10.6) |
| 4 | 48 (11.8) |
| 5 | 30 ( 7.4) |
| 6 | 103 (25.4) |
| mRS 0-2 at 90 days | 182 (44.8) |
| sICH | 34 ( 8.1) |
| NIHSS 24 hours | 9.0 [4.0, 18.0] |
| Increase in the NIHSS>2 at 24 hours | 320 (81.8) |
| Death within 90 days | 103 (25.4) |
| Post-treatment stent occlusion | 51 (12.7) |
| Timing postprocedural stent occlusion |  |
| Within 24 hours | 28 (62.2) |
| 24hours -7days | 14 (31.1) |
| After day 7 | 3 ( 6.7) |
| Any SAH | 80 (19.9) |

**Supplemental** **table 2: Patient outcomes.** mRS: modified Rankin Scale. NIHSS: National Institutes of Health Stroke Scale. SAH: Subarachnoid Hemorrhage. sICH: Symptomatic Intracranial Hemorrhage.

| **SBP variable** | **Minimum** | **Q1** | **Median** | **Q3** | **Maximum** | **Mean** | **Standard deviation** |
| --- | --- | --- | --- | --- | --- | --- | --- |
| Mean SBP | 82,58 | 128,33 | 137,14 | 146,9 | 175,35 | 137,45 | 14,58 |
| Median SBP | 73 | 128 | 137 | 146,5 | 178 | 137,11 | 15,49 |
| Maximum SBP | 105 | 146 | 160 | 173 | 230 | 160,18 | 20,26 |
| Minimum SBP | 15 | 104 | 116 | 130 | 170 | 116,18 | 19,37 |
| Delta SBP | 3 | 25 | 40 | 58 | 131 | 44 | 25,57 |
| SD SBP | 1,52 | 8,47 | 13,02 | 17,33 | 46,69 | 13,82 | 7,37 |
| CV SBP | 0,88 | 6,25 | 9,58 | 12,89 | 52,87 | 10,18 | 5,73 |
| ARV SBP | 1,25 | 7,67 | 11,5 | 15,75 | 65 | 13,06 | 8,35 |
| SV SBP | 2,06 | 9,56 | 14,97 | 19,69 | 65,48 | 16,15 | 9,57 |

**Supplement table 3: SBP variables.** SBP: systolic blood pressure, Q1: 25% percentile, Q3: 75% percentile, SD: standard deviation, CV: coefficient of variation, ARV: average real variability, SV: successive variability.

|  | **aOR** | **95% CI** | **P-value** |
| --- | --- | --- | --- |
| ***sICH*** |  |  |  |
| Mean SBP (per 10 mmHg) | 1.014 | [0.760, 1.358] | 0.925 |
| Median SBP (per 10 mmHg) | 1.024 | [0.778, 1.353] | 0.863 |
| Maximum SBP (per 10 mmHg) | 0.847 | [0.709, 1.030] | 0.072 |
| (Maximum SBP (per 10 mmHg), quadratic term) | 1.000 | [1.000, 1.001] | 0.080 |
| Minimum SBP (per 10 mmHg) | 1.078 | [0.854, 1.371] | 0.531 |
| Maximum difference in SBP over 24hours (per 10 mmHg) | 0.912 | [0.748, 1.092] | 0.339 |
| Standard deviation SBP over 24hours (per 10 mmHg) | 0.918 | [0.479, 1.631] | 0.783 |
| Coefficient of variation SBP over 24hours (per 0.1) | 0.997 | [0.988, 1.006] | 0.575 |
| Average real variability SBP (per 10 mmHg) | 1.043 | [0.609, 1.613] | 0.863 |
| Successive variation SBP (per 10 mmHg) | 0.955 | [0.581, 1.445] | 0.841 |
| ***Death at 90 days*** |  |  |  |
| Mean SBP (per 10 mmHg) | 1.072 | [0.899, 1.282] | 0.439 |
| Median SBP (per 10 mmHg) | 1.079 | [0.915, 1.276] | 0.368 |
| Maximum SBP (per 10 mmHg) | 1.056 | [0.927, 1.201] | 0.409 |
| Minimum SBP (per 10 mmHg) | 0.942 | [0.819, 1.081] | 0.396 |
| Maximum difference in SBP over 24hours (per 10 mmHg) | 1.071 | [0.966, 1.187] | 0.190 |
| Standard deviation SBP over 24hours (per 10 mmHg) | 1.369 | [0.956, 1.961] | 0.085 |
| Coefficient of variation SBP over 24hours (per 0.1) | 1.004 | [0.999, 1.009] | 0.110 |
| Average real variability SBP (per 10 mmHg) | 1.287 | [0.941, 1.749] | 0.107 |
| Successive variation SBP (per 10 mmHg) | 1.236 | [0.941, 1.614] | 0.121 |
| ***Any SAH*** |  |  |  |
| Mean SBP (per 10 mmHg) | 1.171 | [0.967, 1.424] | 0.109 |
| Median SBP (per 10 mmHg) | 1.146 | [0.957, 1.377] | 0.140 |
| Maximum SBP (per 10 mmHg) | 1.036 | [0.910, 1.177] | 0.590 |
| Minimum SBP (per 10 mmHg) | 1.12 | [0.953, 1.323] | 0.174 |
| Maximum difference in SBP over 24hours (per 10 mmHg) | 0.972 | [0.864, 1.087] | 0.623 |
| Standard deviation SBP over 24hours (per 10 mmHg) | 0.96 | [0.636, 1.415] | 0.842 |
| Coefficient of variation SBP over 24hours (per 0.1) | 0.999 | [0.993, 1.004] | 0.613 |
| Average real variability SBP (per 10 mmHg) | 0.898 | [0.622, 1.243] | 0.538 |
| Successive variation SBP (per 10 mmHg) | 0.869 | [0.627, 1.165] | 0.370 |
| ***Increase of NIHSS > 2 at 24hours*** |  |  |  |
| Mean SBP (per 10 mmHg) | 1.248 | [1.008, 1.556] | 0.045 |
| Median SBP (per 10 mmHg) | 1.227 | [0.998, 1.520] | 0.056 |
| Maximum SBP (per 10 mmHg) | 1.216 | [1.037, 1.441] | 0.019 |
| Minimum SBP (per 10 mmHg) | 1.077 | [0.913, 1.272] | 0.378 |
| Maximum difference in SBP over 24hours (per 10 mmHg) | 1.091 | [0.959, 1.251] | 0.198 |
| Standard deviation SBP over 24hours (per 10 mmHg) | 1.616 | [0.991, 2.754] | 0.065 |
| Coefficient of variation SBP over 24hours (per 0.1) | 1.006 | [0.999, 1.013] | 0.113 |
| Average real variability SBP (per 10 mmHg) | 1.192 | [0.803, 1.887] | 0.418 |
| Successive variation SBP (per 10 mmHg) | 1.195 | [0.847, 1.759] | 0.337 |

**Supplement table 3: Adjusted BP association with the secondary outcomes.** aOR: adjusted odds ratio, CI: confidence interval, mRS: modified rankin scale, SBP: systolic blood pressure, sICH: symptomatic intracranial hemorrhage, SAH: subarachnoid hemorrhage, NIHSS: national institutes of health stroke scale.

| **Number of patients** | **Excluded (n=171)** | **Included (n=437)** | **p-value** |
| --- | --- | --- | --- |
| Age | 70.0 (11.9) | 67.1 (13.0) | 0,01 |
| Sex = Male | 107 (62.6) | 260 (59.5) | 0,545 |
| Hypertension | 135 (81.3) | 313 (74.2) | 0,084 |
| Dyslipidemia | 26 (18.7) | 142 (40.0) | <0.001 |
| Diabetes mellitus | 52 (31.9) | 140 (33.5) | 0,789 |
| Coronary artery occlusive disease | 14 ( 8.6) | 65 (16.5) | 0,023 |
| Current or past smoking | 56 (35.7) | 145 (37.0) | 0,848 |
| Atrial fibrillation | 38 (23.3) | 61 (14.7) | 0,018 |
| History of stroke or TIA | 36 (22.1) | 103 (25.7) | 0,429 |
| Pre-stroke mRS |  |  | <0.001 |
| 0 | 71 (41.8) | 312 (75.7) |  |
| 1 | 76 (44.7) | 55 (13.3) |  |
| 2 | 16 ( 9.4) | 24 ( 5.8) |  |
| 3 | 6 ( 3.5) | 17 ( 4.1) |  |
| 4 | 0 ( 0.0) | 4 ( 1.0) |  |
| 5 | 1 ( 0.6) | 0 ( 0.0) |  |

**Supplement table 4: Sensitivity analysis;** Comparison of baseline characteristics of patients included in this analysis (≥four hourly measurements) and those with fewer measurements.

mRS: modified rankin scale; TIA: Transient Ischemic Attack.


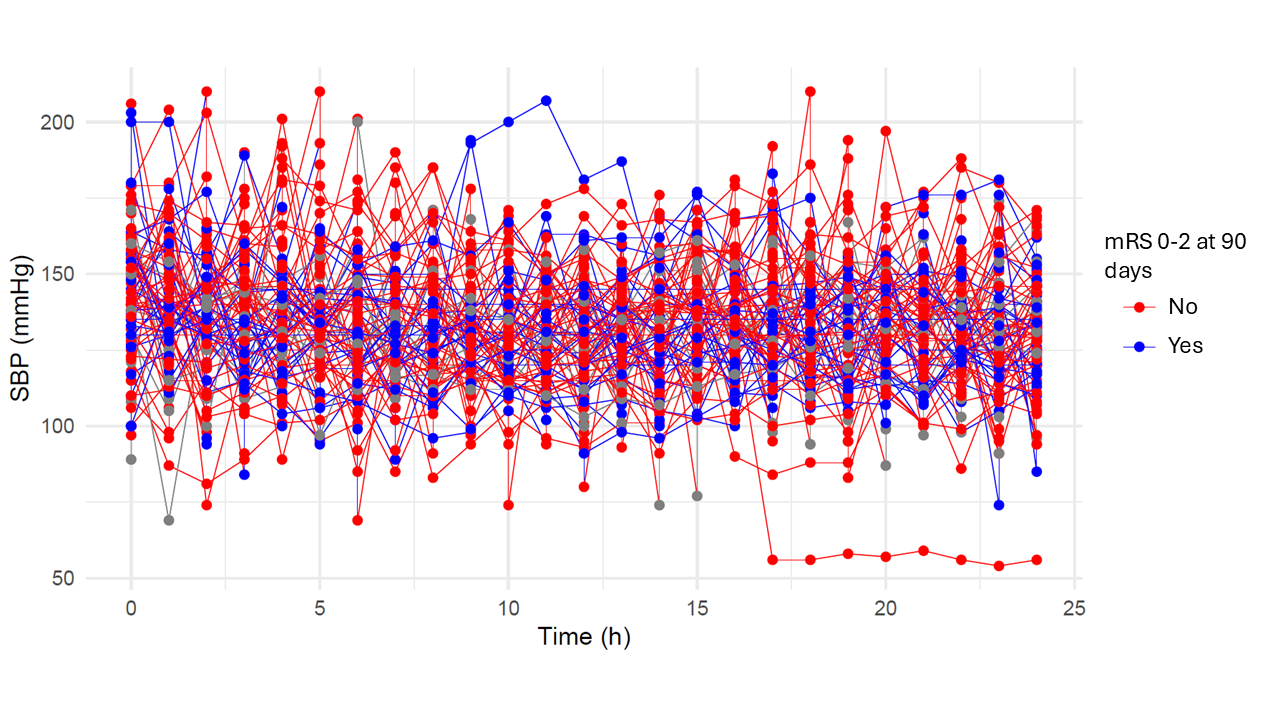


**Supplement figure 2** : course of SBP over 24 hours per patient, stratified by mRS 0-2 at 90 days (1 = yes, 0 = no).
